# Effectiveness and Safety of Perampanel in Refractory Focal Epilepsy: Real‐World Evidence From a Chinese Cohort

**DOI:** 10.1002/brb3.70968

**Published:** 2025-10-29

**Authors:** Tong Yi, Luwen Huang, Wei Peng, Ting Zhang, Dong Zhou, Xiaohui Lai, Jinmei Li

**Affiliations:** ^1^ Department of Neurology West China Hospital of Sichuan University Chengdu Sichuan China; ^2^ Department of Neurology Suining Central Hospital Suining Sichuan Province China; ^3^ Department of Neurology Sichuan Taikang Hospital Chengdu Sichuan China; ^4^ Institute of Brain Science and Brain‐inspired Technology of West China Hospital, Sichuan University Chengdu Sichuan China

**Keywords:** add‐on therapy, effectiveness, perampanel, refractory focal epilepsy, safety

## Abstract

**Objective:**

To evaluate the effectiveness and safety of perampanel (PER) as an add‐on treatment in patients with refractory focal epilepsy.

**Methods:**

This single‐center, retrospective observational study consecutively enrolled patients with refractory focal epilepsy who initiated PER as adjunctive therapy between September 2020 and October 2021. The primary outcomes included PER effectiveness (≥ 50% seizure reduction), treatment retention rates, and adverse event (AE) profiles, systematically evaluated at 3‐, 6‐, and 12‐month follow‐up intervals.

**Results:**

This study included 190 patients (median age 27 years; 51.6% female). At 3/6/12‐month follow‐ups, response rates were 65.5%/64.9%/66.1%, seizure‐free rates 26.2%/26.2%/26.8%, and retention rates 88.4%/69.0%/66.8%, respectively. Logistic regression analysis indicated that females had a lower response rate (OR = 0.38, 95% CI = 0.20.74, *p* = 0.005). Responders had a higher PER dose (≥ 6 mg) than nonresponders at all follow‐ups. Patients receiving late add‐on PER treatment had significantly higher response and seizure‐free rate than those receiving early add‐on therapy. AEs were reported in 41.1% of patients, mostly mild to moderate in severity. The most frequent AEs were dizziness (26.3%), somnolence (13.0%), and psychiatric symptoms (12.11%). Additionally, patients aged ≥ 18 years had a lower risk of AEs (OR = 0.41, 95% CI: 0.19–0.88, *p* = 0.023).

**Conclusions:**

PER demonstrated clinically meaningful efficacy and acceptable safety in refractory focal epilepsy, with sustained response rates and seizure freedom over 12 months. Male gender, higher doses, and late add‐on use predicted better outcomes, while adults experienced fewer adverse events. These real‐world findings support PER's role in treatment‐resistant cases.

## Graphical abstract

This retrospective real‐world study of 190 Chinese patients with refractory focal epilepsy demonstrated that adjunctive PER achieved sustained responder rates (∼66%) and seizure freedom (∼27%) over 12 months, with an acceptable safety profile. Male gender, higher doses, and late add‐on predicted better outcomes, while adults experienced fewer adverse events.

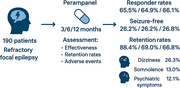

## 1 Introduction

Epilepsy is a major global public health challenge, affecting an estimated 10 million patients in China. Among these patients, focal‐onset seizures account for nearly two‐thirds of cases, with approximately 30% progressing to refractory epilepsy (Ding et al. 2021; Asadi‐Pooya et al. 2023; Brodie et al. 2010). This drug‐resistant form of epilepsy not only severely compromises patients' quality of life but also imposes considerable socioeconomic burdens on both individuals and healthcare systems. For surgical ineligible patients, there remains an urgent unmet need for anti‐seizure medications (ASMs) that demonstrate improved efficacy while maintaining favorable tolerability profiles.

Perampanel (PER) is a third‐generation ASM that functions as a selective, noncompetitive antagonist of AMPA‐type glutamate receptors (AMPAR). Its novel mechanism of action—uniquely targeting postsynaptic glutamate excitability—confers broad‐spectrum therapeutic potential when combined with other ASMs (Hanada 2014). Currently approved in China for patients aged ≥ 4 years, PER is indicated for adjunctive treatment of both focal‐onset seizures (with or without secondary generalization) and primary generalized tonic–clonic seizures (Zhao et al. 2023; Yu et al. 2022; W. R. Zhang et al. 2023; Renfroe et al. 2022; Steinhoff et al. 2013). While international clinical trials and real‐world studies have established PER's favorable efficacy and safety profile in both monotherapy and adjunctive settings (Steinhoff et al. 2013; Chan and Leung 2024; Y. Zhang et al. 2022a; Lim et al. 2021; R. Zhang et al. 2021; Weiping et al. 2021; Liguori et al. 2023; Lossius et al. 2021), comprehensive clinical experience with PER combination therapy remains limited in the Chinese population, particularly regarding long‐term outcomes and optimal dosing strategies.

This real‐world study aimed to comprehensively evaluate PER as adjunctive therapy in refractory focal epilepsy, with three primary endpoints: (a) clinical efficacy (response and seizure‐free rates), (b) treatment retention, and (c) safety profiles. Focusing on a Southwest Chinese population, we conducted detailed subgroup analyses to identify clinically relevant predictors of treatment outcomes, including dose‐response relationships, concomitant ASM effects, and demographic factors. This study represents the first large‐scale real‐world analysis of PER in Southwest China. And, these findings may provide critical regional data to optimize PER utilization in clinical practice.

## 2 Methods

### 2.1 Study Cohort

This hospital‐based retrospective cohort study consecutively enrolled patients with refractory focal epilepsy who initiated adjunctive PER therapy at West China Hospital between September 2020 and October 2021. Refractory epilepsy was strictly defined per ILAE criteria as persistent seizures despite adequate trials of two appropriately selected and tolerated antiseizure medication regimens at therapeutic doses, either as monotherapy or combination therapy (Kwan et al. 2010). Eligible participants met all following criteria: (a) aged ≥ 4 years; (b) definitive diagnosis of focal epilepsy (with/without focal to bilateral tonic–clonic seizures) according to 2017 ILAE classification criteria (Fisher et al. 2017), confirmed by standardized clinical evaluation and ≥ 30‐min EEG recordings using the international 10–20 electrode placement system demonstrating ictal/interictal epileptiform activity; (c) complete baseline data including seizure frequency logs, medication history, and neuroimaging results. We excluded patients using any herbal preparations with purported antiepileptic effects (e.g., gastrodin, *Uncaria rhynchophylla*).

This retrospective observational study was approved by the Ethics Committee of West China Hospital, Sichuan University (No: 2020732). Informed consent was obtained from all individual participants included in the study.

### 2.2 PER Treatment

PER was administered orally as adjunctive therapy in patients with refractory focal epilepsy, following a standardized titration protocol: treatment was initiated at 2 mg once daily at bedtime, with dose increments of 2 mg every 2 weeks based on individual tolerance and therapeutic response, up to a maximum dose of 12 mg/day (Y. zhang et al. 2022b). Pediatric patients (4–17 years) were initiated at 2 mg qHS with slower titration (generally +2 mg every 3–4 weeks), whereas most adults followed the +2 mg every 2 weeks schedule when tolerated. The final maintenance dose was individualized according to each patient's seizure control and adverse effect profile, following the principle of optimal risk‐benefit balance.

### 2.3 Data Collection

All patients included in this study were independently reviewed by two neurologists. Data were collected using a structured case report form, including demographic data, epilepsy‐related clinical information (e.g., age at seizure onset, epilepsy duration, etiology, baseline seizure frequency), use of ASMs before and during PER treatment, reasons and timing for discontinuation, and AEs. Baseline seizure frequency was defined as the average seizure frequency (times per month) during the 6 months preceding the addition of PER. Each patient was required to maintain a seizure diary, primarily recorded by their caregivers, to inform clinicians about seizure occurrences. Patients visited the clinic with a caregiver at scheduled evaluation time points. Outcome parameters were recorded at each visit until October 2021, the date of death, or the date of the last clinic visit if lost to follow‐up. Based on the patient's self‐report, caregiver's description, and seizure diary, an epilepsy specialist documented the number of seizures, frequency, detailed seizure course, PER dose, type and dose of concomitant ASM, and any post‐medication discomfort (absent/present, with specific symptoms).

### 2.4 Assessment of Efficacy and Safety

The primary endpoint was the proportion of responders at 3‐, 6‐, and 12‐month follow‐ups. Secondary endpoints included the retention rate and drug‐related AEs at 3‐, 6‐, and 12‐month follow‐ups. Patients who achieved a ≥ 50% reduction in monthly seizure frequency compared to the baseline phase were classified as responders. The baseline frequency was defined as the mean seizure frequency (times/month) for the 6 months before the addition of PER. Seizure freedom was defined as complete seizure control with PER since the previous visit. For the 12‐month visit, seizure freedom was defined as no seizure in the preceding 6 months; for the 3‐ and 6‐month visits, it referred to no seizures since baseline or the 3‐month visit, respectively. The retention rate was defined as the proportion of patients who continued PER treatment at the time of assessment. The safety analysis set included all patients who received at least one dose of PER. Safety was assessed based on the type and frequency of all AEs and withdrawals related to PER from drug initiation to the last follow‐up.

### 2.5 Subgroup Validation

Two types of add‐on PER were categorized as early add‐on (≤ 2 prior ASMs) and late add‐on (≥ 3 prior ASMs) (Liguori et al. 2023). Patients receiving carbamazepine, oxcarbazepine, phenytoin, or topiramate, which can affect PER plasma concentration (Rohracher et al. 2016; Villanueva, Majid, et al. 2016), were classified into the concomitant enzyme inducers group. Structural and nonstructural epilepsy were defined according to the presence of a lesion on MRI.

### 2.6 Statistical Analysis

Continuous data were presented as mean ± standard deviation (mean ± SD) or median ± interquartile range (median ± IQR) and analyzed using the independent samples *t*‐test. Categorical data were presented as frequencies and percentages (*n*, %), and differences between groups were compared using the Pearson chi‐square test.

All patients were included in the intention‐to‐treat analysis. For instance, patients who discontinued PER treatment for any reason after 3 months were categorized as nonresponders during the 6‐ and 12‐month follow‐up periods. Univariate analysis and multivariate logistic regression were conducted to identify predictors for responders and seizure‐free at follow‐ups. Logistic regression was also used to identify risk factors for AEs. The results were presented as adjusted odds ratios (ORs) and 95% confidence intervals (CIs). Statistical analysis was performed using R 3.3.2 (accessible at http://www.R‐project.org, The R Foundation), Free Statistics software version 1.4, and GraphPad Prism 8.0. A *p* < 0.05 was considered statistically significant.

## 3 Results

### 3.1 Demographics and Baseline Characteristics

A total of 190 patients were included in the study. Baseline demographics and clinical characteristics are detailed in Table S1. The median age was 27.0 years (IQR: 20.0–34.0), and 98 patients (51.6%) were female. A total of 36 patients (18.9%) had a structural etiology, while 130 (68.4%) had epilepsy of unknown origin. Additionally, 67 patients (35.3%) were on three or more concomitant ASMs, with the most commonly used being levetiracetam (62.1%), oxcarbazepine (47.9%), and valproic acid (34.7%) (Figure S1). Regarding background therapy, children received fewer concomitant ASMs than adults in our dataset (median 2 vs. 3 agents). The risk of certain adverse events (AEs), particularly gait disturbance and dizziness, increases with the number of concomitant ASMs (OR 5.15–5.85 and 1.85–1.86, respectively), while overall AE incidence and somnolence show less clear trends.

**FIGURE 1:**
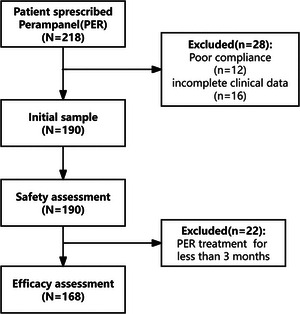
Patient flow.

A total of 22 patients discontinued treatment within the first 3 months due to AEs, leaving 168 patients for the effectiveness analysis (see patient flow in Figure 1). At the 12‐month follow‐up, the median maintenance dose of PER in 122 patients was 6 mg (range: 2–12 mg; mean: 5.4 ± 1.8 mg) (Figure S2).

### 3.2 Retention Rate and Responder Rate

The retention rates at 3, 6, and 12 months were 88.42%, 68.95%, and 66.84%, respectively. The reasons for patient withdrawal and retention at follow‐ups were shown in Figure S3, with lack of efficacy being the most common reason for withdrawal (38 patients, 54.29%), followed by the presence of AEs (18 patients, 25.71%).

**FIGURE 2:**
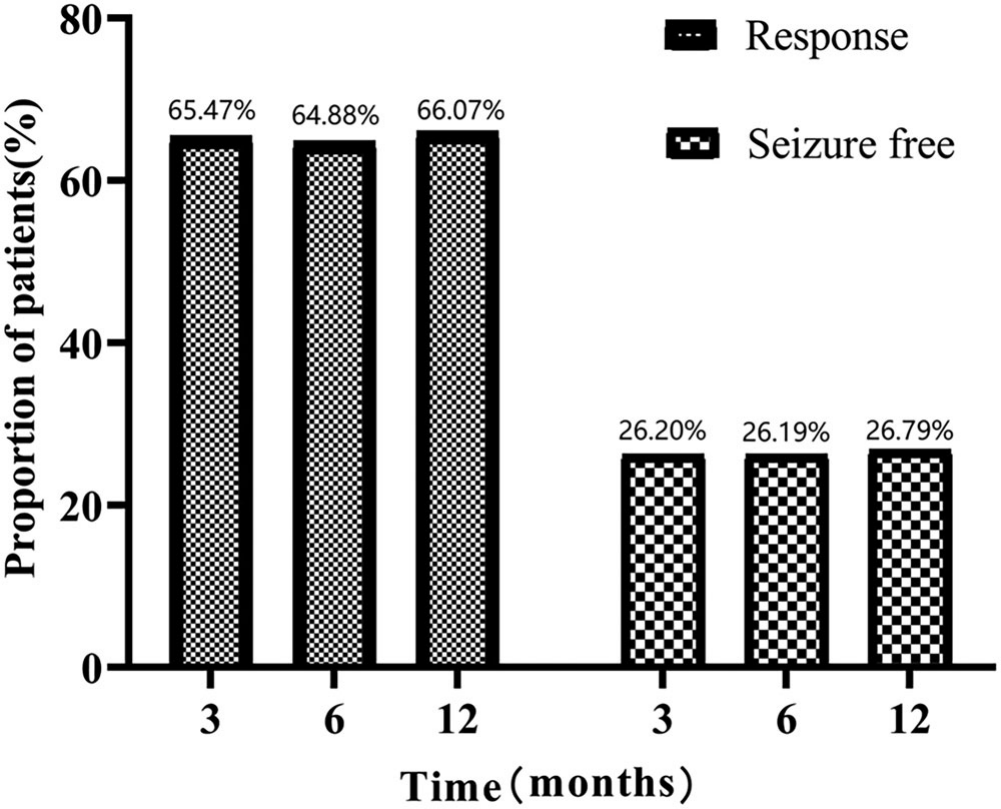
Efficacy of PER with follow‐up time.

The proportions of responders at 3, 6, and 12 months were 65.47%, 64.88%, and 66.07%, respectively, while seizure freedom at these time points was 26.20%, 26.19%, and 26.79% (Figure 2). Univariate analysis revealed that response to PER was significantly associated with gender, epilepsy duration, maintenance dose, and late add‐on PER (Table 1).

**TABLE 1:** Univariate analysis for variables associated with nonresponders/responders and non‐seizure‐free/seizure‐free.

Variables	3 months (*N* = 157)			6months (*N* = 153)	12months (*N* = 156)	
	Responders (*N* = 110)	Seizure free (*N* = 47)	Responders (*N* = 109)	Seizure free(*N* = 44)	Responders (*N* = 111)	Seizure free (*N* = 45)
**Age, *n***						
< 18 years (*n* = 35)	20 (64.52%)	10 (32.26%)	21 (67.74%)	10 (32.26%)	23 (74.19%)	9 (29.03%)
≥ 18 years (*n* = 155)	90 (65.69%)	37 (27.01%)	88 (64.23%)	34 (24.82%)	88 (64.23%)	36 (26.28%)
*p*	0.9	0.55	0.71	0.4	0.29	0.75
**Gender, *n***						
Female (*n* = 98)	50 (58.14%)	20 (23.26%)	46 (53.49%)	19 (22.09%)	48 (55.81%)	22 (25.58%)
*p*	0.04	0.16	0.02	0.22	0.005	0.72
**Dose, mg, *n***						
≤ 4 mg (*n* = 97)	42 (56.00%)	22 (29.33%)	39 (52.00%)	21 (28.00%)	41 (54.67%)	20 (26.67%)
≥6 mg (*n* = 92)	68 (73.91%)	25 (27.17%)	70 (76.09%)	23(25.00%)	70 (76.09%)	25 (27.17%)
OR (95 CI%)	2.23(1.16, 4.27)	0.90(0.46,1.77)	2.94(1.52,5.68)	0.86(0.43, 1.71)	2.64(1.36, 5.11)	1.03(0.52, 2.04)
*p*	0.02	0.76	0.001	0.66	0.004	0.94
**Etiology, *n***						
Nonstructural (*n* = 154)	87 (63.97%)	36 (26.47%)	88 (64.71%)	34 (25.00%)	90 (66.18%)	39 (28.68%)
Structural (*n* = 36)	23 (71.88%)	11 (34.38%)	21 (65.62%)	10(31.25%)	21 (65.62%)	6 (18.75%)
OR (95 CI%)	1.44(0.62, 3.36)	1.46(0.64,3.31)	1.04(0.46, 2.34)	1.36(0.59,3.7)	0.98(0.43,2.20)	0.57(0.22,1.50)
*p*	0.3991	0.3718	0.9219	0.4705	0.9527	0.2582
**Late add‐on, *n***						
No (*n* = 92)	29 (40.85%)	12 (16.90%)	24 (33.80%)	7 (9.86%)	29 (40.85%)	8 (11.27%)
Yes (*n* = 98)	81 (83.51%)	35 (36.08%)	85 (87.63%)	37(38.14%)	82 (84.54%)	37 (38.14%)
OR (95 CI%)	7.33 (3.59,14.99)	2.78 (1.32, 5.85)	13.87 (6.36,30.24)	5.64 (2.34,13.61)	7.92 (3.83,16.36)	4.86 (2.09,11.27)
*p*	< 0.001	0.007	< 0.001	< 0.001	< 0.001	< 0.001
**Secondary GTCS, *n***						
No (*n* = 113)	62 (61.39%)	27 (26.73%)	62 (61.39%)	25 (24.75%)	64 (63.37%)	23 (22.77%)
Yes (*n* = 77)	48 (71.64%)	20 (29.85%)	47 (70.15%)	19 (28.36%)	47 (70.15%)	22 (32.84%)
OR (95 CI%)	1.59 (0.82, 3.09)	1.17 (0.59, 2.31)	1.48 (0.76, 2.86)	1.20 (0.60, 2.42)	1.36 (0.70, 2.63)	1.66 (0.83, 3.31)
*p*	0.17	0.66	0.25	0.60	0.36	0.15

Patients with a maintenance dose of ≥ 6 mg were more likely to respond compared to those on lower doses across the 3‐, 6‐, and 12‐month follow‐ups (OR = 2.23, 95% CI: 1.16–4.27, *p* = 0.016) (Table 1). Late add‐on PER was associated with drug response at 3, 6, and 12 months (OR = 7.33, 95% CI: 3.59–14.99, *p* < 0.001; OR = 13.87, 95% CI: 6.36–30.24, *p* < 0.001; and OR = 7.92, 95% CI: 3.83–16.36, *p* < 0.001, respectively). This factor was also linked to seizure freedom at 3 months (OR = 2.78, 95% CI: 1.32–5.85, *p* = 0.007), 6 months (OR = 5.64, 95% CI: 2.34–13.61, *p* < 0.001), and 12 months (OR = 4.86, 95% CI: 2.09–11.27, *p* < 0.001). Patients receiving late add‐on PER had a median epilepsy duration of 9.0 years (IQR: 5.0–12.0), compared to 10.0 years (IQR: 7.0–20.0) for those with early add‐on PER (Table S2). At the 3‐month follow‐up, males showed a higher response rate than females (OR = 1.96, 95% CI: 1.03–3.76, *p* = 0.04), a trend that persisted at 6 months (OR = 2.88, 95% CI: 1.48–5.61, *p* = 0.02) and 12‐months (OR = 2.62, 95% CI: 1.35–5.11, *p* = 0.005).

### 3.3 Safety and Tolerability

During the follow‐up, AEs were reported in 78 patients (41.1%). The most frequent AEs were dizziness (50, 26.3%), somnolence (25, 13.2%), and gait disturbance (20, 10.5%) (Table 2 and Figure 4). Psychiatric AEs included emotional disorders in 15 patients (7.9%), irritability in 8 patients (4.2%), and suicidal tendencies in 1 patient (0.5%). Eighteen patients (9.5%) discontinued PER due to AEs, primarily due to emotional disorders (6, 3.1%) and irritability (4, 2.1%).

**TABLE 2:** Treatment‐emergent adverse effects.

Symptom	Total, *n* (%)
Any AE, *n* (%)	78 (41.1)
Withdrawal due to AEs, *n* (%)	20 (10.5)
Severity of TEAE	
Mild, *n* (%)	32 (16.8)
Moderate, *n* (%)	29 (15.3)
Severe, *n* (%)	17 (8.9)

Abbreviations: AEs, adverse events; *n*, number of patients; TEAE, Treatment‐Emergent Adverse Event.

Patients aged ≥ 18 years exhibited a significantly lower risk of AEs even after adjusting for potential risk factors (OR = 0.41, 95% CI = 0.19–0.88, *p* = 0.023) (Table 3). This negative association was particularly evident for dizziness (OR = 0.38, 95% CI: 0.17–0.85, *p* = 0.018). Conversely, taking three or more concomitant ASMs was positively associated with experiencing gait disturbances (OR = 5.85, 95% CI: 2.04–16.78, *p* = 0.001). Gender, a maintenance dose of ≥ 4 mg, and the use of concomitant enzyme inducers were not significantly associated with PER‐related AEs.

**TABLE 3:** Regression analysis for risk factors of AEs.

Characteristic	AEs (OR, 95% Cl)		Dizziness (OR, 95% Cl)		Somolence (OR, 95% Cl)		Gait disturbance (OR, 95% Cl)	
	Model 1	Model 2	Model 1	Model 2	Model 1	Model 2	Model 1	Model 2
Female^#^	1.17 (0.65–2.08)	1.24 (0.68–2.26)	1.42 (0.74–2.73)	1.49 (0.76–2.94)	0.85 (0.37–1.97)	1.1 (0.46–2.64)	0.59 (0.23–1.52)	0.6 (0.23–1.6)
Age ≥ 18 years*	**0.39 (0.18**–**0.82)**	**0.41 (0.19**–**0.88)**	**0.39 (0.18**–**0.84)**	**0.38 (0.17**–**0.85)**	**0.33 (0.13**–**0.83)**	0.45 (0.17–1.17)	0.89 (0.28–2.85)	0.93 (0.28–3.11)
Maintenance dose ≥ 4 mg	0.94 (0.52–1.69)	0.89 (0.48–1.66)	1.42 (0.74–2.72)	1.49 (0.76–2.91)	1.17 (0.5–2.71)	1.35(0.56–3.22)	1.19 (0.46–3.08)	1.21 (0.46–3.18)
Concomitant ASMs (*n* ≥ 3)	1.15 (0.63–2.11)	1.12 (0.6–2.06)	1.86 (0.96–3.6)	1.85 (0.95–3.63)	0.68 (0.27–1.72)	0.7 (0.27–1.8)	5.15 (1.88–14.14)	5.85 (2.04–16.78)
Concomitant enzyme inducer	0.66 (0.33–1.31)	0.66 (0.33–1.32)	1.05 (0.48–2.28)	1.02 (0.47–2.25)	1.62 (0.53–5.02)	1.74 (0.55–5.55)	2.86 (0.64–12.85)	2.88 (0.64–13.02)

*Note*: Model 1: Crude OR (95% Cl); Model 2: #Adjusted for age, sleep disturbance, and mood disorders at baseline and duration of epilepsy. ^a^Adjusted for gender, sleep disturbance, and mood disorders at baseline and duration of epilepsy. The other three groups were adjusted for age, gender, sleep disturbance, and mood disorders at baseline and duration of epilepsy.

Abbreviations: AEs, adverse events; ASM, anti‐epileptic drugs.

## 4 Discussion

This study analyzed the effectiveness and safety of adjunctive PER in treating refractory focal epilepsy in Southwest China, using a sample size of 190 patients. The findings provide real‐world insights into the use of PER, confirming its effectiveness and safety. PER add‐on therapy significantly improved seizure outcomes in patients with focal refractory epilepsy.

Notably, in this study, 66.07% of patients (*n* = 111) achieved a positive response at the last follow‐up, a figure higher than the 26%–56% reported in randomized controlled trials (Lossius et al. 2021; Krauss et al. 2012; French et al. 2012) and the 23%–52.3% reported in other real‐world studies (Y. Zhang et al. 2022a; Lossius et al. 2021; Steinhoff, Bacher, et al. 2014; Ishikawa et al. 2019). The proportion of patients achieving seizure freedom (26.79%) was comparable to those in the Austrian study (27%) (Rohracher et al. 2016) and the WRAPPER study (25.6%) (Steinhoff et al. 2013), although it was slightly higher than the 14%–18.1% reported in other real‐world investigations (Y. Zhang et al. 2022a; Steinhoff, Bacher, et al. 2014; Steinhoff, Hamer, et al. 2014). Several factors may account for these variations. First, differences in patient demographics, including baseline seizure frequency, epilepsy classification, and previous treatments, may exist among studies. For example, in a Chinese clinical trial where PER was used as adjunctive therapy, 47.2% of patients showed drug response (Y. Zhang et al. 2022a). However, that study included patients with both focal and generalized epilepsy, suggesting a potentially lower response rate in populations with generalized epilepsy. Additionally, the severity of drug resistance influenced the prognosis. In our study, only 0.6% of patients experienced Grade III resistance (after six or more AED failures) (Gomez‐Alonso and Gil‐Nagel 2010), compared to 16.7% in the study by Zhang et al., which may explain the lower response rate in the latter. Second, the duration of follow‐up could have affected the results. In the WRAPPER study, the seizure freedom rate increased with longer follow‐up, reaching 12.9%, 20.7%, and 25.6% at 16, 26, and 52 weeks, respectively. In contrast, other studies observed patients for only 6 months (Y. Zhang et al. 2022a; Steinhoff, Bacher, et al. 2014; Steinhoff, Hamer, et al. 2014). Furthermore, some patients received additional ASMs, vagus nerve stimulation, or surgery after add‐on PER, potentially improving their prognosis (Raspin et al. 2023).

The 12‐month retention rate in our study (66.84%) was lower than the 77.8%–80.5% reported in other studies (Y. Zhang et al. 2022a; Toledano Delgado et al. 2020; Abril Jaramillo et al. 2020). This lower rate could partly be attributed to poor access to PER, with around 13% of withdrawals due to availability issues. Improving access to PER in Southwest China, a developing region, could benefit patients with epilepsy in this area.

Previous studies have found that males have a higher retention rate with PER (Jacob et al. 2017, Bonnett et al. 2012), suggesting better tolerability and fewer AEs in males (Wehner et al. 2017). Canevini et al. (2010) reported that females had higher scores on the AE Profile questionnaire, indicating a greater susceptibility to AEs. Females were also more likely to discontinue treatment due to AEs (Janmohamed et al. 2021), such as cosmetic side effects. Although our study's results were not statistically significant, they were consistent with these findings (retention rate: female [*n*, %] vs. male [*n*, %] = 41, 58.57% vs. 29, 41.43%, *p* = 0.14). In our cohort, weight gain was the predominant cosmetic concern (overall ∼5%), and several withdrawals among women cited weight gain as a primary factor. Evidence supports PER‐associated weight gain, with associations to serum PER concentration and vulnerabilities such as intellectual disability. Recent Chinese real‐world data also list weight gain among TEAEs. Reports and meta‐analyses of PER real‐world safety similarly include weight gain alongside dizziness, somnolence, gait issues, and behavioral symptoms. Therefore, for patients experiencing such AEs, clinicians should provide tailored counseling on lifestyle modifications, including dietary and physical activity guidance, consider bedtime dosing to minimize daytime fatigue, and adopt slower titration schedules if early weight gain emerges.

Asian patients tend to receive lower PER dosage, with studies reporting mean doses of 3.7 mg in Japan (Inoue et al. 2022), 4.3 mg (Wang et al. 2022), or 4.96 mg in China (Y. Zhang et al. 2022a), and 4.39 mg in Korea (Kim and Oh 2018). The median dose in our cohort was 4.0 mg (4.0, 6.0), which was lower than that reported in real‐world studies of non‐Asian populations, such as 6.9 mg in Spain (Villanueva, Garcés, et al. 2016) and 8 mg in Australia. Our data suggest that higher doses may result in better responses. However, Asians generally do not tolerate high doses (10–12 mg/day) well (Tsai et al. 2019). In our study, only three patients (1.58%) achieved a dosage of 10 mg or higher. We hypothesize that higher doses within the tolerated range (< 10 mg) may yield better responses without increasing AEs. Given the differences in pharmacogenomic metabolism and body weight across populations, more data are needed to determine the optimal PER dosage for Chinese patients.

Several studies have shown that the early PER administration is more effective than late add‐on (starting PER treatment after ≥ 3 previous ASMs) (Liguori et al. 2023; Villanueva, Garcés, et al. 2016; Fernandes et al. 2021), as seizure control efficacy decreases with each subsequent ASM addition (Mohanraj and Brodie 2006). However, this does not fully explain PER's efficacy in controlling refractory epilepsy (Y. Zhang et al. 2022a; Rohracher et al. 2016; Juhl and Rubboli 2016). Our findings suggest that PER is more effective as a late add‐on treatment, possibly due to shorter epilepsy duration in the late addition group. Long‐term epilepsy can lead to neuronal loss and mossy fiber sprouting, forming excitatory recurrent circuits that increase drug resistance (Mohanraj and Brodie 2013). To address potential early discontinuation among patients receiving late add‐on PER, a sensitivity analysis was performed, classifying those who discontinued before the 6‐ or 12‐month endpoints as nonresponders. At 6 months, the responder rate decreased slightly from 87.6% to 82.5%, and the seizure‐free rate decreased from 38.1% to 35.1%. At 12 months, the responder rate decreased from 84.5% to 79.4%, and the seizure‐free rate decreased from 38.1% to 34.0% (data not shown). Logistic regression analyses under this conservative assumption indicated that late add‐on PER remained effective, although the magnitude of efficacy was slightly attenuated compared with the primary analysis. These results suggest that early discontinuation may influence observed treatment outcomes in real‐world cohorts and highlight the importance of considering retention bias in effectiveness assessments.

Finally, we note that enzyme‐inducing ASMs (e.g., carbamazepine, phenytoin, oxcarbazepine) can increase PER clearance and lower exposure, sometimes prompting higher clinical doses to achieve the effect. In our cohort, 79.2% of patients were on enzyme‐inducing ASMs, with a mean PER dose of 5.4 ± 1.8 mg, higher than the overall median of 4 mg, which may also contribute to observed differences in efficacy and tolerability.

The concurrent use of enzyme‐inducing ASMs may negatively impact the response to PER treatment. Alexandra et al. (Rohracher et al. 2016) found that patients using enzyme inducers had lower seizure freedom rates than those who did not. However, consistent with our findings, a prospective observational study from Germany (Steinhoff, Bacher, et al. 2014) found no significant difference in outcomes between patients using enzyme inducers and those who did not. Enzyme inducers, such as carbamazepine, oxcarbazepine, and phenytoin, can interfere with PER metabolism, leading clearance and decreasing serum PER concentrations (Rohracher et al. 2016; Villanueva, Majid, et al. 2016). Carbamazepine, the most common enzyme inducer among ASMs, reduces PER blood concentration by approximately 70% (Gidal et al. 2013). Nevertheless, PER showed significant superiority over placebo even when coadministered with carbamazepine (Steinhoff et al. 2013). US prescribing guidelines note that co‐administration of ≥ 4 mg PER with carbamazepine may mitigate the enzyme‐inducing effect (Food & Drug Administration, 2012) (Steinhoff et al. 2013). In our study, patients using enzyme inducers had a mean PER dose of 5.3 ± 1.9 mg, higher than the 4 mg mean dose for all patients. Additionally, only 8.42% of patients in our study were using carbamazepine, which may have influenced the observed outcomes. Prospective studies with larger sample sizes are needed to confirm these conclusions.

PER was well‐tolerated in our cohort, with an AE rate of 41.1% during follow‐up, compared to 50%–67.6% in previous observational studies (Steinhoff, Bacher, et al. 2014; Shah et al. 2016; Huber and Schmid 2017; Villanueva et al. 2018). Most AEs in our study were mild to moderate and did not necessitate discontinuation of PER. Logistic regression analysis identified several meaningful risk factors. First, patients younger than 18 years were more likely to experience AEs, indicating better tolerance in adults. This finding aligns with previous studies (Swiderska et al. 2017; Heyman et al. 2017), suggesting that slower titration and lower PER doses should be considered for pediatric patients. Second, consistent with previous studies (Villanueva et al. 2018; Rohracher et al. 2018), AE incidence was not correlated with PER dosage, suggesting that the dosages used were appropriate for this population. Finally, a higher risk of gait disturbance was observed in patients using ≥ 3 concomitant ASMs, indicating that greater caution is needed for these patients, such as considering much slower titration (e.g., 2 mg every 4 weeks) (Youn et al. 2018).

The most common AEs of PER in our study were dizziness, somnolence, emotional disorders, and irritability, consistent with findings from other real‐world studies (French et al. 2012; Fogarasi et al. 2020; French et al. 2015). Dizziness and somnolence are common side effects of many ASMs, including lacosamide, zonisamide, and pregabalin (Martyn‐St James et al. 2012; Zaccara et al. 2008). These AEs can often be minimized by taking the PER before bedtime or adjusting the dose (Steinhoff, Bacher, et al. 2014). The incidence of psychiatric AEs in this study (12.11%) was within the expected range for refractory epilepsy patients (Lossius et al. 2021; Rohracher et al. 2016; Toledano Delgado et al. 2020). Psychiatric AEs, such as hostility and aggression, have been found to increase at higher PER doses in pooled Phase III study analysis (Steinhoff et al. 2013). Despite the lower mean dose of PER in our study, we did not observe a lower incidence of psychiatric AEs. Therefore, psychiatric symptoms should be carefully monitored in patients with refractory epilepsy, particularly those with psychiatric comorbidities or on multiple ASMs. Behavioral and psychiatric adverse events (PBAEs) are not unique to PER. PBAEs—including irritability, aggression, mood lability, anxiety, and psychosis—have been reported with several ASMs. Topiramate exhibits multiple mechanisms, including AMPA/kainate receptor antagonism, and psychiatric AEs are well documented (e.g., irritability/aggression). Levetiracetam is not an AMPA antagonist; its primary mechanism is SV2A binding, but behavioral AEs (irritability, agitation, mood changes) are well recognized. We now explicitly clarify the mechanistic distinction (AMPA antagonism for PER; SV2A modulation for LEV) while acknowledging the clinical overlap of behavioral AEs across ASMs, as reviewed by recent PBAE analyses. This addition contextualizes our behavioral AE findings within the broader ASM landscape and helps guide monitoring and counseling. Prior real‐world and pediatric reports also note similar AE spectra but emphasize that slower titration in younger patients can mitigate behavioral and somnolence events. This highlights the importance of cautious up‐titration and careful monitoring of pediatric patients, particularly given the limited sample size in our cohort.

This study has several limitations. First, the data were collected retrospectively, which may introduce biases inherent to such study designs. Second, although the sample size was larger than in many other studies, the number of patients aged ≥ 65 years was limited (2, 1.05%), which restricts the generalizability of the findings to older populations. Third, the absence of data on PER blood concentrations made it impossible to assess the impact of enzyme‐inducing ASMs on PER levels, limiting our ability to fully evaluate potential drug interactions.

## 5 Conclusion

In conclusion, the study confirmed the effectiveness and tolerability of PER add‐on therapy for patients with focal refractory epilepsy in Southwest China. The dosage in this cohort was lower than that in non‐Asian populations, yet the responder rate was comparable. Patients who took higher doses had better responses without a higher incidence of AEs. AEs; were associated with the use of at least three concomitant ASMs but not with PER dosage.

## Author Contributions

**Tong Yi**: Data curation, Formal analysis, Visualization, Writing – original draft. **Luwen Huang**: Methodology, Data curation, Statistical analysis, Writing – review & editing. **Wei Peng**: Patient recruitment, Data collection, Investigation. **Ting Zhang**: Patient recruitment, Data collection, Investigation. **Dong Zhou**: Supervision, Conceptualization, Clinical guidance.**Xiaohui Lai**: Study design, Supervision, Writing – review & editing. **Jinmei Li**: Conceptualization, Supervision, Project administration, Writing – review & editing.

## Ethics Statement

This retrospective observational study was approved by the Ethics Committee of West China Hospital, Sichuan University (No: 2020732).

## Consent

All patients signed informed consent for participation in this study.

## Conflicts of Interest

The authors declare no conflicts of interest.

## Peer Review

The peer review history for this article is available at https://publons.com/publon/10.1002/brb3.70968

## Supporting information




**Supporting Fig.1**: The condition of concomitant AEDs in total population.

**Supporting Fig.2**: The condition of varying PER dosages in the total population.

Supplemental Fig. 3 Reasons for PER Treatment Discontinuation.

Supplemental Fig. 4 Treatment‐emergent adverse effects associated with the central nervous system, psychiatric and gastrointestinal.

Supplemental table 1 Clinical characteristics of patients using PER.

Supplemental table 2 The condition of early add‐on and late add‐on PER.

## Data Availability

The data that support the findings of this study are available from the corresponding author upon reasonable request.
