# Supplementary figures and images for: Effectiveness and Safety of Perampanel in Refractory Focal Epilepsy: Real‐World Evidence From a Chinese Cohort
Source: Brain Behav. 2025 Oct 29;15(11):e70968. doi: 10.1002/brb3.70968 (PMC12571969; doi:10.1002/brb3.70968)

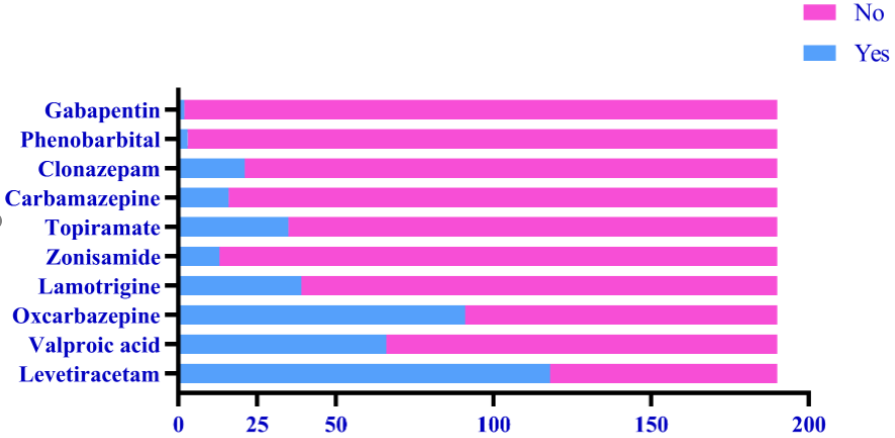

Supplement: Supplementary file 1 — Supporting Fig.1: The condition of concomitant AEDs in total population. [file BRB3-15-e70968-s005.png]

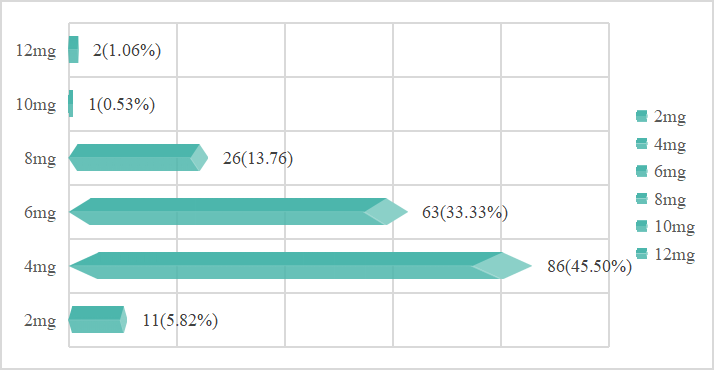

Supplement: Supplementary file 2 — Supporting Fig.2: The condition of varying PER dosages in the total population. [file BRB3-15-e70968-s003.png]

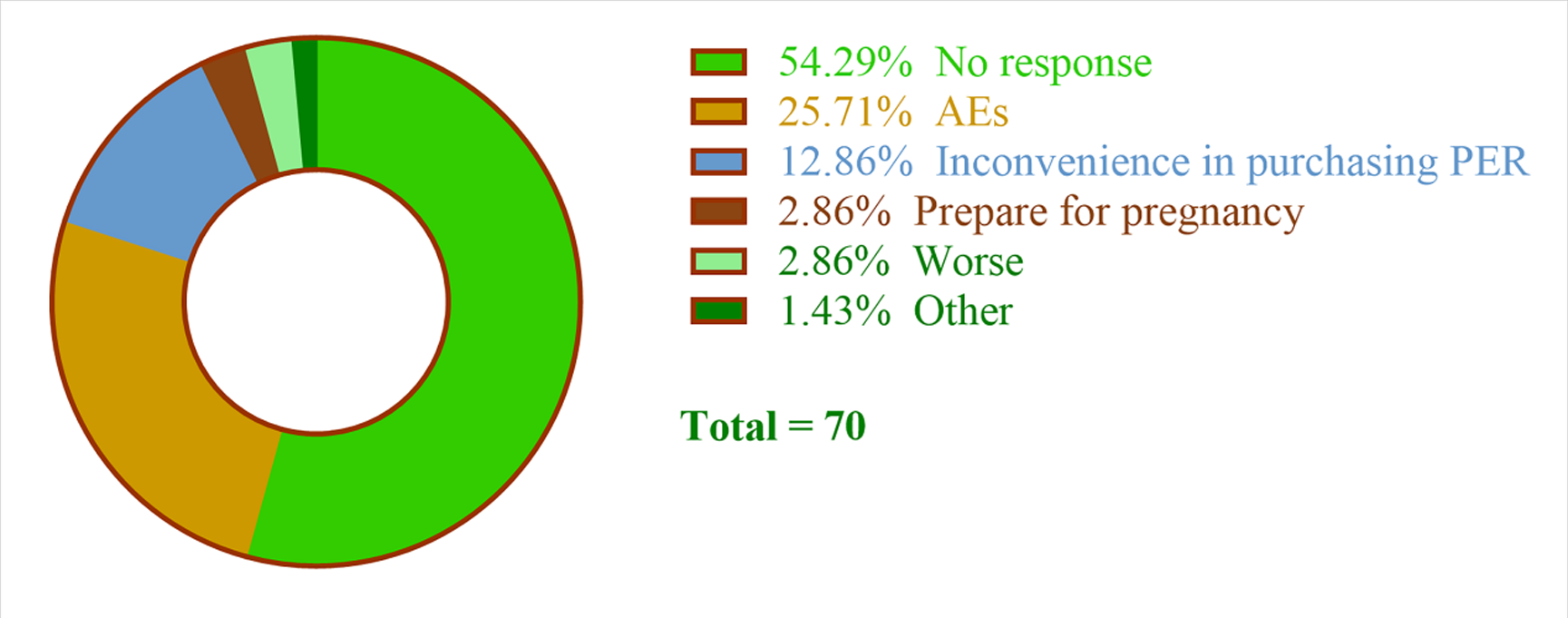

Supplement: Supplementary file 3 — Supplemental Fig. 3 Reasons for PER Treatment Discontinuation. [file BRB3-15-e70968-s002.tif]

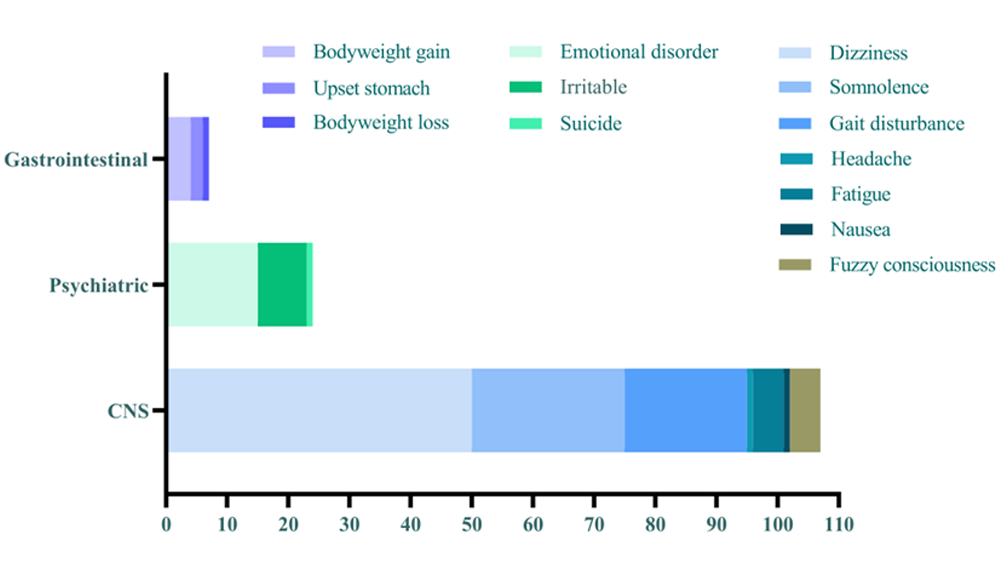

Supplement: Supplementary file 4 — Supplemental Fig. 4 Treatment‐emergent adverse effects associated with the central nervous system, psychiatric and gastrointestinal. [file BRB3-15-e70968-s001.png]
