# Supplementary material for: Effectiveness and Safety of Perampanel in Refractory Focal Epilepsy: Real‐World Evidence From a Chinese Cohort
Source: Brain Behav. 2025 Oct 29;15(11):e70968. doi: 10.1002/brb3.70968 (PMC12571969; doi:10.1002/brb3.70968)
Supplement: Supplementary file 5 — Supplemental table 1 Clinical characteristics of patients using PER. [file BRB3-15-e70968-s006.docx]

Supplementary Table 1 Clinical characteristics of patients using perampanel

| **Characteristic** | **Total population**  **(n = 190)** | **Effectiveness population**  **(n = 168)** |
| --- | --- | --- |
| **Female gender, n (%)** | 98 (51.6) | 86 (51.2) |
| **Age, years, Median (IQR)** | 27.0 (20.0, 34.0) | 27.0 (19.8, 35.0) |
| **Age group, years, n (%)** |  |  |
| 4≤n＜18 | 35 (18.4) | 31 (18.5) |
| n≥18 | 155 (81.6) | 137 (81.5) |
| **Duration of epilepsy, years, median (IQR)** | 10.0 (6.0, 15.0) | 10.0 (6.0, 15.0) |
| **Duration of epilepsy, years, n (%)** |  |  |
| ≤10 | 121 (63.7) | 109 (64.9) |
| ＞10 | 69 (36.3) | 59 (35.1) |
| **Etiology of epilepsy, n (%)** |  |  |
| Structural | 36 (18.9) | 32 (19.0) |
| No structural | 154 (81.1) | 136 (81.0) |
| **Symptomatic epilepsy, n (%)** |  |  |
| Parietal lobe | 1 (0.5) | 1 (0.6) |
| Frontal lobe | 2 (1.1) | 2 (1.2) |
| Occipital lobe | 3 (1.6) | 1 (0.6) |
| Temporal lobe | 44 (23.2) | 42 (25.0) |
| Unknown | 140 (73.7) | 122 (72.6) |
| **Origin of epilepsy, n (%)** |  |  |
| Multifocal | 15 (7.9) | 15 (8.9) |
| focal | 45 (23.7) | 41 (24.4) |
| Unknown | 130 (68.4) | 112 (66.7) |
| **Awareness, n (%)** |  |  |
| No | 173 (91.1) | 153 (91.1) |
| Yes | 17 (8.9) | 15 (8.9) |
| **Aura, n (%)** |  |  |
| No | 118 (62.1) | 106 (63.1) |
| Yes | 72 (37.9) | 62 (36.9) |
| **Motor symptoms, n (%)** |  |  |
| No | 51 (26.8) | 45 (26.8) |
| Yes | 139 (73.2) | 123 (73.2) |
| **Secondary GTCS, n (%)** |  |  |
| No | 113 (59.5) | 101 (60.1) |
| Yes | 77 (40.5) | 67 (39.9) |
| **Epileptogenic MRI lesion, n (%)** |  |  |
| No | 69 (45.7) | 57 (42.5) |
| Yes | 82 (54.3) | 77 (57.5) |
| **Previous AEDs, n (%)** |  |  |
| ＜3 | 78 (41.1) | 65 (38.7) |
| ≥3 | 112 (58.9) | 103 (61.3) |
| **Concomitant AEDs , n (%)** |  |  |
| ＜3 | 123 (64.7) | 105 (62.5) |
| ≥3 | 67 (35.3) | 63 (37.5) |
| **Late add-on, n (%)** |  |  |
| No | 92 (48.4) | 71 (42.3) |
| Yes | 98 (51.6) | 97 (57.7) |
| **Dose, mg, (Mean ± SD)** | 5.2 ± 1.8 | 5.4 ± 1.8 |
| **Dose, mg, median (IQR)** | 4.0 (4.0, 6.0) | 6.0 (4.0, 6.0) |
| **Maintenance Dose, mg, n (%)** |  |  |
| ≤4mg | 97 (51.3) | 75 (44.9) |
| ≥6mg | 92 (48.7) | 92 (55.1) |
| **Concomitant enzyme inducer, n (%)** |  |  |
| No | 43 (22.6) | 35 (20.8) |
| Yes | 147 (77.4) | 133 (79.2) |

Abbreviations: AED, antiepileptic drug; IQR, interquartile range; SD, standard deviation;
