# Supplementary material for: Effectiveness and Safety of Perampanel in Refractory Focal Epilepsy: Real‐World Evidence From a Chinese Cohort
Source: Brain Behav. 2025 Oct 29;15(11):e70968. doi: 10.1002/brb3.70968 (PMC12571969; doi:10.1002/brb3.70968)
Supplement: Supplementary file 6 — Supplemental table 2 The condition of early add‐on and late add‐on PER. [file BRB3-15-e70968-s004.docx]

**Supplementary Table 2 The condition of early add-on and late add-on PER**

| **Variables** | **Early add-on**  **(n = 71)** | **Late add-on**  **(n = 97)** | ***p*** |
| --- | --- | --- | --- |
| **Gender** |  |  | 0.838 |
| Male | 34 (47.9) | 48 (49.5) |  |
| Female | 37 (52.1) | 49 (50.5) |  |
| **Awareness, n (%)** |  |  | 0.363 |
| No | 8 (11.3) | 7 (7.2) |  |
| Yes | 63 (88.7) | 90 (92.8) |  |
| **Aura, n (%)** |  |  | 0.061 |
| No | 39 (54.9) | 67 (69.1) |  |
| Yes | 32 (45.1) | 30 (30.9) |  |
| **Motor symptoms, n (%)** |  |  | 0.72 |
| No | 18 (25.4) | 27 (27.8) |  |
| Yes | 53 (74.6) | 70 (72.2) |  |
| **Secondary to GTCS, n (%)** |  |  | 0.044 |
| No | 49 (69) | 52 (53.6) |  |
| Yes | 22 (31) | 45 (46.4) |  |
| **Previous AEDs, n (%)** |  |  | 0.637 |
| ＜3 | 26 (36.6) | 39 (40.2) |  |
| ≥3 | 45 (63.4) | 58 (59.8) |  |
| **Maintainece Dose, mg, n (%)** |  |  | 0.007 |
| ≤4mg | 40 (57.1) | 35 (36.1) |  |
| ≥6mg | 30 (42.9) | 62 (63.9) |  |
| **Age, years, Median (IQR)** | 29.0 (21.5, 37.0) | 26.0 (18.0, 33.0) | 0.107 |
| **Duration of epilepsy, years, median (IQR)** | 10.0 (7.0, 20.0) | 9.0 (5.0, 12.0) | 0.014 |
